# Simian virus 40 in humans

**DOI:** 10.1186/1750-9378-2-13

**Published:** 2007-07-09

**Authors:** Fernanda Martini, Alfredo Corallini, Veronica Balatti, Silvia Sabbioni, Cecilia Pancaldi, Mauro Tognon

**Affiliations:** 1 Department of Morphology and Embryology, Section of Cell Biology and Molecular Genetics, School of Medicine, and Center of Biotechnology, University of Ferrara, Via Fossato di Mortara, 64/B. 44100 Ferrara, Italy; 2 Department of Experimental and Diagnostic Medicine, Section of Microbiology, University of Ferrara, Via Luigi Borsari, 46. 44100 Ferrara, Italy

## Abstract

Simian virus 40 (SV40) is a monkey virus that was administered to human populations by contaminated vaccines which were produced in SV40 naturally infected monkey cells.

Recent molecular biology and epidemiological studies suggest that SV40 may be contagiously transmitted in humans by horizontal infection, independently from the earlier administration of SV40-contaminated vaccines.

SV40 footprints in humans have been found associated at high prevalence with specific tumor types such as brain and bone tumors, mesotheliomas and lymphomas and with kidney diseases, and at lower prevalence in blood samples from healthy donors.

Contrasting reports appeared in the literature on the circulation of SV40 in humans by contagious transmission and its association, as a possible etiologic cofactor, with specific human tumors. As a consequence of the conflicting results, a considerable debate has developed in the scientific community. In the present review we consider the main results obtained by different groups investigating SV40 sequences in human tumors and in blood specimens, the putative role of SV40 in the onset/progression of specific human tumors, and comment on the hypotheses arising from these data.

## Background

Simian virus 40 (SV40) is a monkey virus which was accidentally administered to humans, in the years 1955-'63, through contaminated poliovirus vaccines. The subsequent findings of the transforming and oncogenity activities of the SV40 viral large T (Tag) and small t (tag) antigens, have prompted investigations into the potential of SV40 to induce cancer in humans. To date, hundreds of molecular and epidemiologic studies aimed at investigating whether SV40 infects humans, its potential mode of transmission and its putative role in human tumors, have been published.

In this review we evaluate the biological, pathological and clinical evidence of SV40 in human cancers, non-malignant diseases and blood samples.

### Simian virus 40

SV40 was assigned to the family of *Papovaviridae*, an acronym proposed by Melnick and obtained by fusing the names of the three representative viruses *Pa*pilloma, *Po*lyoma, and *Va*cuolating agent. More recently, SV40 has been considered a Polyomavirus, together with the human BK (BKV) and JC (JCV) Polyomaviruses. The virion is about 45 nm, an icosahedral particle, with a density of 1.34–1.35 g/cm3. The viral genome is a circular, double-stranded DNA molecule. SV40 codes, at least, for six viral proteins: two early nonstructural polypeptides, the large tumor antigen (Tag) and the small tumor antigen (tag), an agnoprotein, probably involved in the assembly of viral particles and processing of late mRNA and three capsid proteins, VP1, VP2, and VP3 [1-3]. The early and late genes are transcribed on different DNA strands in a way that the transcription proceeds divergently from the regulatory region. This region contains the origin of DNA replication and binding sites for the transcription factors that control viral gene expression, and terminates within DNA sequences containing the polyadenylation signals. Recently, a predicted late polarity pre-microRNA (pre-miRNA) to the untranslated region 3' of the polyadenylation cleavage site in the late pre-mRNA has also been detected [4,5].

SV40 is phylogenetically closely related to the human JCV and BKV. They evidence similarity with respect to size (~5.2 Kb), genome organization and DNA sequence. The Tags of SV40, BKV and JCV strongly cross-react with the same antisera [6,7], while a less strong cross-reactivity is observed in most structural antigenic determinants of the viral proteins, named VP1, 2 and 3. A genus-specific capsid antigen, located on viral peptide VP1, has been identified [8]. The DNA sequences of SV40 share 70% homology with BKV [9], and 69% with JCV [10]. The greatest homology is found in the early region coding for the Tags and tags, whereas a lower homology is detected in the regulatory region.

### SV40 Life Cycle in Host Cells

The cell infection starts by the binding of the SV40 virus to a receptor on the cell membrane. This receptor has been identified as the major histocompatibility complex (MHC) [11]. In monkeys, initial lytic infection by SV40 is controlled by the immune system. Then, SV40 persisting infection occurs in the kidney cells where it may be reactivated by immunosuppression [12]. The life cycle of SV40 in humans is poorly understood [13]. After binding to the cell surface, polyomavirus capsid undergoes endocytosis and is transported to the nucleus where the viral DNA is uncoated and transcription of the early region begins. SV40 enters cells by caveola-mediated endocytosis [14]. The primary transcript from the early region is alternatively spliced to give two mRNAs that encode large Tag and small tag. Tag is a nuclear phosphoprotein of 94 kD and it is an essential factor for viral DNA replication. It binds to the viral origin of replication (ori) where it promotes unwinding of the double helix and recruitment of cellular proteins that are required for DNA synthesis, including DNA polymerase-α and replication protein A [15-18]. SV40 relies on cellular enzymes and cofactors for DNA replication and these proteins are expressed in S phase. Tag modulates cellular signaling pathways to induce cells to enter S phase and this accounts for the ability of Tag to transform cells. Large Tag is thought to stimulate the cell cycle through its ability to bind to several cellular proteins that are involved in crucial signal transduction pathways that control cell cycle progression and apoptosis [19]. The role of the small tag in the polyomavirus life cycle is less clear. Analysis of SV40 deletion mutants revealed that tag is not essential for lytic infection in culture [20]. However, tag cooperates with Tag in the transformation of cells by SV40 [21,22] and increases virus yield in permissive cells [23]. As viral replication proceeds, the late genes begin to be expressed. In permissive cells, Tag acts to stimulate transcription from the late promoter and represses transcription from the early promoter. The gene products of the late region are the capsid proteins VP1, VP2, and VP3, which assemble with the replicated viral DNA to form virions, which are released upon cell lysis. SV40 DNA can become integrated into the chromosomal DNA of the cell especially upon infection of non-permissive cells [24]. Integration occurs at random both in terms of the site in the cellular genome and in the viral genome [25].

Different studies indicate that SV40 can replicate productively in human cells, including spongioblasts, fetal neural cells, newborn kidney cells [26], and some tumor cell lines [27,28], although it grows poorly in human fibroblasts [29]. Some human cell types undergo visible cell lysis in response to SV40 infection, whereas other cells fail to exhibit cytopathic changes and produce low virus levels [29]. Human mesothelial cells do not support the lytic infection efficiently and are transformed at a high rate (1,000 times higher than that of human fibroblasts) [30] by SV40 or Tag alone [22,23], and release SV40 virions as a result of the persistent infection [30,31]. The same behavior has been observed in human lymphoblastoid B-cell lines where SV40 progeny is produced at a low level [32]. This behavior peculiar to SV40 in specific human cells, such as mesothelial cells, is uncommon for a DNA tumor virus. Indeed, it is well established that DNA tumor viruses either infect permissive cell lines with the production of an infectious viral progeny or infect and transform non-permissive cells without a productive viral cycle.

### SV40 Cell Transformation and Experimental Tumorigenesis

Transformation of rodent and human cells by SV40 is induced by the two oncoproteins, the large Tag and the small tag, which display multiple functions. The main activity of Tag for cell transformation and tumorigenesis is to target key cellular proteins, such as the tumor suppressor p53 and pRB family proteins, inactivating their functions [13,19,33-40]. SV40 Tag may also lead to transformation by inducing mutations to the cellular genome [41] or numerical and structural alterations of chromosomes [42,43], such as gaps, breaks, dicentric and ring chromosomes, chromatid exchanges, deletions, duplications and translocations [44].

The principal role of the small tag in transformation is to bind the catalytic (36 kDa) and regulatory (63 kDa) subunits of protein phosphatase 2A (PP2A) [13,19], inactivating their function. Moreover, tag interacts with the centrosome and blocks mitosis in human cells [45], suggesting that it may disrupt cell cycle progression. Recently, it has been shown that in human mammary epithelial cells tag activates phosphatidylinositol 3-kinase [46], an enzyme involved in pathways crucial for cell proliferation and transformation. In addition, SV40 tag is able to enhance transcription from E2F-activated promoters of early growth response genes [47,48].

The process of rodent cell transformation induced by SV40 typically depends on the integration of the viral DNA into the host genome where it produces a high level of expression of the major viral oncogenic proteins, large T antigen and small t antigen [13,19,33-36]. However, human cells experimentally transformed by SV40 harbor viral genomes in an episomal state, sometimes in great number, in addition to integrated viral DNA [13,19,33-36].

SV40 immortalized and transformed human cells [49-52] are able to induce tumors when implanted subcutaneously in autologous hosts [51]. In some cases, SV40 Tag needs cooperation of the catalytic subunit of telomerase and the activated c-H-*ras *oncogene, for the complete transformation of human cells, as shown in cotransfection experiments [53].

SV40 is highly oncogenic in rodents and when inoculated subcutaneously, intracerebrally or intravenously in newborn hamsters induces soft tissue sarcomas, osteosarcomas, ependymomas and choroid plexus papillomas, and neoplasms of the hematopoietic system, such as lymphocytic leukemia, histiocytic lymphomas and rarely, B-cell lymphomas [33,54-56]. Moreover, direct inoculation of SV40 into the pleural space induces malignant mesothelioma in 100% of the injected hamsters [55]. The oncogenic potential of SV40 is confirmed by the generation of transgenic mice in which polyomavirus large Tag expression is regulated by the native viral early promoter-enhancer [57]. SV40-transgenic mice, like rodents experimentally inoculated with the virus, develop ependymomas and choroid plexus papillomas, as well as other neoplasms [33,58-60].

### Epidemiology of SV40 infection in humans

SV40 natural infection in humans is considered a rare event, restricted to people living in contact with monkeys, the natural hosts of the virus, such as inhabitants of Indian villages located close to the jungle, and workers attending to monkeys in zoos and animal facilities [61,62]. A source of human exposure to SV40 occurred between 1955 and 1963, when inactivated and live anti-polio vaccines, prepared from polioviruses grown in naturally SV40-infected simian cell cultures, were administered to hundreds of millions of people in the United States, Canada, Europe, Asia and Africa [63]. Soon it was shown that children vaccinated with contaminated oral polio vaccines (OPV) shed infectious SV40 in stools for at least 5 weeks after vaccination [64]. However, some children, who received the same OPV, did not develop neutralizing antibodies even though they may have received large doses of live SV40, compared with the potentially inactivated SV40 in inactivated polio vaccine (IPV). Further, SV40 human contamination occurred in experimental infection with live respiratory syncytial virus to adult volunteers and a neutralizing antibody response in about two thirds of the volunteers was shown [65]. Inactivated vaccines against adenoviruses [66] and hepatitis A [67] virus also exposed humans to SV40, although the amount of infectious SV40 was almost certainly lower then that administered with OPV or live respiratory syncytial virus.

Early serologic studies reported the presence of SV40 neutralizing antibodies, at different titles, in the population that received IPV. Immune response appeared to correlate with the amount of SV40 present in the vaccine; 30% to 50% of individuals reached a significant antibody response against formalin-inactivated SV40 after three doses of the vaccine. Antibody titles persisted for a period of up to 3 years post-inoculation [68]. Additional serologic studies reported a SV40 seropositivity in individuals with no history of immunization with contaminated IPV or other possible route of SV40 infection [69-72]. Shah et al., [72] detected antibodies to SV40 in children born after 1964, when IPV was free of SV40, as well as in people born before 1954. These studies suggest that humans may become infected by SV40 independently from poliovirus vaccine exposure. However, most of these early serologic studies were carried out before the discovery of the two human polyomaviruses, BK and JC, which are close related to SV40 and are ubiquitous in human populations. It is possible that the early serologic evidence of SV40 antibody detection in human sera represents some degree of cross reactivity with antibodies against the highly related BK and JC viruses [73-75].

To date, the prevalence of SV40 infections in humans is not known. Recent studies, based on PCR and serological techniques, indicate that SV40 infection occurs both in children and adults. (i) SV40 DNA sequences have been detected in normal and neoplastic tissues of people either too young (1 to 30 years) or too old (60 to 85 years) to have been vaccinated with SV40-contaminated anti-polio vaccines [19,33,76-81]. This finding may also explain the lack of difference in cancer incidence between individuals vaccinated with SV40-contaminated and SV40-free anti-polio vaccines [82]. (ii) SV40 sequences and Tag were detected in blood and sperm specimens from normal individuals and oncologic patients [80,81,83-88] and in lymphoblastoid cells [32]. These results suggest that PBMCs could be a reservoir and vehicle of SV40 spreading in the tissues of the host and among the individuals. (iii) SV40 sequences were found in urine and stoole samples, from children and adults [84,89,90], indicating that the haematic, sexual and orofecal routes of transmission are likely to be responsible for SV40 horizontal infection in humans. (iv) Infectious SV40 was rescued by transfection of permissive CV-1 monkey cells with the DNA of an SV40-positive human choroid plexus carcinoma, one blood and one HPV-infected normal vulvar tissue samples [91,92]. Finally, specific antibodies to SV40 capsid antigens have recently been found in human sera [93-104]. Unfortunately there is no comparative data on the SV40 DNA prevalence in PBMC and antibodies presence to SV40 antigens in sera from the same patients.

Among recent SV40 serologic investigations, some data suggest that specific SV40 antibodies are present in human sera: (i) in a collection of human sera from Morocco, 100% of the samples had antibodies to SV40 [97], whereas in the same study other sera from Morocco, Zaire, Sierra Leone and Poland contained SV40 antibodies in 0.4 to 5.3% of the samples [97], a figure which corresponds with the results of other surveys [93,101]. All the sera in the 100% positive Moroccan collection were from cases of poliomyelitis in children under five years of age [97]. These children, therefore, had probably not been vaccinated against poliovirus. This result should not be overestimated, especially because the collection of the 100% anti-SV40-positive sera was made up of only 29 samples. Nevertheless, this observation suggests that, under particular circumstances, humans can display a great specific antibody response to SV40. Perhaps, the overt poliomyelitis syndrome has influenced the immunological reaction of affected patients to SV40. (ii) Although it is obviously difficult, due to the ubiquity of the two human polyomaviruses, to find a human serum positive for SV40 and negative for both BKV and JCV antibodies, one such serum was detected [100]. (iii) While seroconversion to BKV and JCV is age-dependent [105,106], there is no data on the age-dependent seroconversion to SV40 [100,101], suggesting that most of the SV40 antibodies present in human sera are not generated by infection with BKV or JCV. (iv) In sera from two immunosuppressed renal transplant patients, that were examined sequentially for antibodies to BKV, JCV and SV40 over a period of 82 and 51 weeks, respectively, a significant rise in SV40 antibody titers was detected [97], indicating that a latent SV40 infection, like BKV and JCV latent infection, can be reactivated in humans by immunosuppression. Moreover, during the post-transplant follow-up, the evolving profile of antibodies to SV40 was clearly different from that of antibodies to either BKV or JCV [97], suggesting a specific immunological response to SV40 in these two patients.

Some other recent SV40 serological studies, using virus neutralization and ELISA test, showed antibodies to SV40 in a limited number (1.3 to 15.6%) of normal human sera, suggesting a low virus circulation in the human population [102-104,107]. Other studies have examined seroprevalence for SV40 in cancer patients compared with controls using viral-like-particle (VLP)- based assays, which detect antibodies that are specific to the major capsid protein VP1 of SV40, BKV, and JCV [97-104,107]. These studies showed that SV40 seroprevalence is similar between cancer patients compared with controls (ranging from 5% to 10%) and suggest that no association exists between SV40 seroprevalence and either immunization with poliovirus vaccine or cancer incidence [99,100,104].

The antigenic cross-reaction of SV40 with the two human polyomaviruses BK and JC has been, so far, the most difficult problem in studying the real diffusion of SV40 infection in humans. Carter et al. [108], using recombinant SV40 VP1 virus-like particles (VLPs) as antigen in an ELISA test, detected antibodies to SV40 in 6,6% of human sera, whereas SV40 reactivity in the same sera disappeared after serum pre-adsorption with BKV and JCV VLPs. Due to the different results obtained before and after pre-adsorption with BKV and JCV VLPs, this study suggests that most of the seropositivity for SV40 is actually caused by cross reactivity to BKV or JCV [93-100]. The authors concluded that authentic SV40 antibodies are absent in human sera and therefore SV40 does not appear to be a prevalent human pathogen. However, some considerations can be made on the validity of SV40 studies using VLPs. Due to the sequence homology of the VP1 structural proteins (i.e., more than 80% identical) in the three polyomaviruses, BKV, JCV, and SV40, it is not surprising that the vast majority of the human antibody repertoire against the VP1 protein of the three polyomaviruses can largely overlap. This cross-reactivity, in turn, increases the probability that immunodominant epitopes are present within the family of VP1 antigenic determinants common to all three polyomaviruses. Coexistence of immunodominance and cross-reactivity has been largely documented in the literature [109]. Thus, both qualitative and quantitative differences in the antibody response to the epitopes present only in the SV40 VP1 structural protein may be difficult to determine, particularly if an assay based on serum preadsorption with BKV and JCV VLP is used as the sole means of detection. Therefore, due to the great homology of the VP1 structural protein in the three polyomaviruses, preadsorption with BKV and JCV VLPs may have removed from human sera most of the SV40 antibodies, which cross-reacted with human polyomavirus capsid antigens. Furthermore, it is worth noting that ELISA tests can detect non-neutralizing antibodies and some cross-reactivity is not unexpected among the three polyomaviruses. In theory, VLPs should resemble native virions and retain some of their immunological features. However, polyomaviruses VLPs based assays used the VP1 capsid protein, a highly homologous structural protein among the three viruses. Altogether these considerations suggest that the validity of SV40 studies using VLPs assays is problematic [110].

The site of SV40 latent infection in humans is unknown. Detection of SV40 in human kidney and urine [84,85,89] points to the kidney as a site of virus latency, like in the natural monkey host [111,112]. Clues to the mechanisms of SV40 transmission in humans may come from studies of the natural infection in monkeys [112]. Since uninfected weaning animals do not frequently seroconvert when grouped with the infected mothers or littermates, it seems most likely that transmission of SV40 in monkeys, under conditions of natural infection, occurs after weaning from the environment rather than directly from other animals [112]. Interestingly, this observation would support SV40 transmission in humans from the contaminated general environment or from the home environment [90]. Finally, SV40, as with other tumor viruses, tends to establish long-term persistent infection, as compared with the self-limited infection typical of most common viruses [113]. Therefore, the response of the host exerts constant pressure on chronic virus infection and, in defence, viruses contain genes that have the potential to modulate such host responses. Indeed, recent data indicate that SV40 miRNAs downregulate Tag, a target of the cytotoxic T lymphocyte (CTL) response expression, promoting the CTL evasion in "in vivo" conditions [4,5]. Thus, down-regulating the accumulation of unnecessary Tag, the SV40 miRNAs reduce CTL susceptibility and local cytokine release. Although this down-regulation is dispensable for viral growth in culture, it is likely to be of considerable importance "in vivo". Predicted hairpin structure for the pre-miRNA is not only found in all SV40 isolates, but also conserved in other primate polyomaviruses, including BKV and JCV [4,5].

### SV40 in human non-malignant specimens

SV40 sequences were detected in kidney and cells of urine sediments from patients with focal segmental glomerulosclerosis and SV40 was isolated by co-cultivation of cells from urine sediments of such patients with CV-1 monkey cells [84,85]. SV40 DNA was localized to renal tubular epithelial cell nuclei in renal biopsies of patients with focal segmental glomerulosclerosis as determined by in situ hybridization. Several strains of SV40 were rescued in this study, including strain 776 and other strains bearing mutations in the early and late regions [85]. One study reported that SV40 and BKV sequences have been co-detected in the kidneys of patients with post-transplantation interstitial nephritis thus suggesting that SV40 may cooperate in the etiopathogenesis of this chronic disease [84]. Moreover, the presence of SV40 in kidney tissue [84,85] and urine [89] points to the kidney as a site of virus latency, like in the natural monkey host [111,112]. Other studies showed that SV40 DNA sequences from the viral regulatory region were detected and identified in the allografts of immunocompromised pediatric renal transplant recipients and in the native kidneys of a young adult lung transplant patient with polyomavirus nephropathy [94,114]. Different studies have detected SV40 DNA sequences in PBMCs from various patient populations [32,80,81,83,85,88]. These results demonstrate the nephrotropic and lymphotropic properties of SV40 and indicate that the kidney can serve as a reservoir for the virus in humans. It appears that patients with acquired and/or iatrogenic immunosuppression are a population at risk for SV40. However, the frequency, natural history, and morbidity of the virus in this increasing patient population are unclear.

### Association of SV40 with human tumor specimens

SV40 sequences have been found, mainly by PCR methods, in different human cancers including mesothelioma, osteosarcoma, and non-Hodgkin's lymphoma and some different lymphoproliferative disorders, a variety of childhood brain tumors such as ependymoma and choroid plexus tumors, as well as thyroid, pituitary and parotid gland tumors [32,76-81,87,115-140]. These human tumors correspond to the neoplasms that are induced by SV40 experimental inoculation in rodents [33] or by generation of transgenic mice with the SV40 early region gene directed by its own early promoter-enhancer [57-59]. SV40 sequences were detected in most cases by PCR. However, in two independent studies SV40 sequences were detected, by Southern blot hybridization, integrated in human osteosarcomas and thyroid tumors [130,131]. In addition, infectious SV40 was isolated from a choroid plexus carcinoma as well as from one blood sample and one HPV-infected vulvar tissue sample [91,92]. Two independent studies, conducted in human brain tumor samples [80] and kidney biopsies from patients affected by post-transplantation nephritis [85], showed co-detection of SV40 and BKV sequences in the same sample, suggesting a possible co-operation/helper function between the two polyomaviruses in human cells. However, other studies examining polyomaviruses in brain cancers using primers capable of detecting the three polyomaviruses have not reported co-infection [76,115,117,126,135]. Different types of lymphomas and other lymphoproliferative disorders, from immunosuppressed/HIV+ patients, tested positive for SV40 sequences and Tag expression [87,119,120]. However, the SV40 prevalence in lymphomas from immunosuppressed/HIV+ and HIV-negative oncologic patients did not differ substantially. The semi-permissiveness of human cells to SV40 infections may explain the restricted multiplication of the virus even in the immunosuppression condition of the host.

Some of the above mentioned studies also demonstrated the messenger RNA of SV40 Tag by RT-PCR and/or the viral oncoprotein presence by immunohistochemistry in tumor tissues [32,76,129]. On the contrary, other studies failed to demonstrate both SV40 sequences and Tag protein using similar technical approaches in the same tumor kind [122,141-144]. It has been suggested that different techniques employed in DNA extraction and purification from human specimens may account for the SV40-negative data [87]. Indeed, some popular commercial kits do not allow the recovery of the small SV40 DNA (5.2 kb) when present in a low amount and in the episomal state [81]. In addition, it has been reported that SV40 DNA sequences could be amplified by PCR with certain sets of primers but not with others [76,77,122]. These discrepancies have led to questions regarding the sensitivity and specificity of PCR-based detection of SV40 as well as the possibility of false-positive results caused by laboratory contamination. To settle this dispute, two multi-institutional studies were performed to examine the presence of SV40 in human malignant mesotheliomas [136,142]. Unfortunately, the two investigations reached opposite conclusions, leaving the question unresolved.

Recently, Lopez-Rios et al., [145] raised the possibility that false-positive results could be amplified from common laboratory vectors which contain one or more of SV40 viral DNA elements. Further, Manfredi et al., [146] have failed to detect SV40 sequences in their tumor specimens. These authors call into question all previous studies that have used PCR methods to detect SV40 in human tumors, because they were conducted with primers that can amplify DNA present in common laboratory plasmids. The issue of possible laboratory contamination of specimens is not a new argument. It should be pointed out that, in the recent years, rigorous precautions have been taken in most studies. It is reasonable to suppose that, if SV40-based plasmid contamination occurred during sample processing, it would affect a very large number of samples and frequency of SV40 detection by PCR in human tumors would be higher than that reported. In this context, it should borne in mind that in recent investigations SV40 Tag mRNA and/or Tag protein have been detected both in fresh and paraffin-embedded tissues [76,107,129,137]. Moreover, in human specimens sequence variability in the SV40 Tag C-terminal coding region and regulatory region have been detected [79,125-130,147,148], thus adding additional support to the circulation of different SV40 strains in humans. Indeed, many reports indicate that different SV40 strains and variants are distributed throughout the human population and consequently in human specimens [149]. Variations in the Tag-C gene region have frequently been detected in human tumor associated sequences [79,150,151]. The Tag-C sequence was shown to be stable during the tissue culture passage of SV40 isolates [150]. In contrast, the SV40 regulatory region may contain large insertions, deletions or duplications [151], and rearrangements have been observed to occur within individual infected monkeys [152] and during the passage of SV40 in certain cultured cells [153]. Several SV40 genotypes from monkeys, contaminated vaccines and humans, are common to different population sources. Interestingly, studies based on known isolates and genomic fragments indicate that monkeys, vaccine and human populations contain SV40 genotypes both with one and two 72-bp repeats in the enhancer domain of the regulatory region. Studies derived from the United States mainly detected one 72-bp repeat in human tumor tissues although SV40 regulatory region sequences with two 72-bp repeats were also detected in human osteosarcoma and mesothelioma samples [78,151]. Moreover, SV40 wild type strain 776, which has two 72-bp repeats in the enhancer domain of the regulatory region, was the main representative among the different SV40 strains detected in kidney, urine and blood samples of an American group consisting of normal people and patients affected by focal segmental glomerulosclerosis [84]. In a recent study carried out in Italy, SV40 sequences were detected in peripheral blood lymphocytes from Caucasian organ donors of different ages [154]. Interestingly, the SV40 regulatory region detected in these human specimens showed DNA sequence variability. This result confirms and extends previous data on the circulation of different SV40 strains and variants in different populations. In addition, the presence of SV40 sequences in people born before and after the introduction of SV40-contaminated vaccine suggests that (i) SV40 is spreading by horizontal infection and probably (ii) other unidentified sources of SV40 infection may exist [19].

It has been shown that some vaccine-derived SV40 genotypes overlap with those detected in monkey and in human populations, supporting the hypothesis that contaminated vaccines may play a role in the introduction of SV40 into the human population [155]. Moreover, it should be noted that viral isolates from humans with one 72-bp in the regulatory region have not diverged from monkey isolates, showing that adaptation is not essential for viral survival in humans [155].

As mentioned above, SV40 sequences in human tumors were often detected at low level, less than one genome equivalent per cell [87], while the Tag expression when detected was revealed only in a fraction of tumor cells [87]. These data obtained with human specimens differ from the results from rodent cells, where the SV40 sequences are present in each cell and the SV40 Tag must be continuously expressed to transform the cell and then to maintain transformation. In order to understand, at least in part, these differences between human and rodent models, some considerations should be made. It is well established that the SV40 Tag induces chromosome aberrations [42,43] which are likely to affect the functions of genes involved in tumorigenesis, such as oncogenes, tumor suppressor and DNA repair genes [155-158]. Once chromosomal damage has been triggered in tumors and chromosomal aberrations have reached a threshold, genomic instability ensues [158], due to the functional alteration of DNA repair genes, leading to more genetic lesions and tumor progression [158,159]. This process does not need the long-period maintenance of the original oncogenic agent that caused the injury and initiated the tumorigenesis process. Therefore, in human cells SV40 could initiate the tumorigenic process by hitting the cell genome, then it could become dispensable and lost in the progression phase of the tumor, when the accumulation of genetic alterations renders the presence of viral transforming functions unnecessary. Immunoselection may even be exerted against persistently SV40-infected cells, while genetically mutated and uninfected cells may have a proliferative advantage and become the prevalent population in the tumor tissue. This "hit and run" mechanism was originally proposed to explain transformation of human cells by the mutagenic herpesviruses [160,161], and has been recently suggested to be effective in colorectal carcinogenesis associated with JC virus, a polyomavirus closely related to SV40 [162]. On the contrary, in SV40-transformed rodent cells, SV40 sequences are not lost. This difference mainly depends on rodent cells, which are non-permissive to SV40 multiplication. Therefore, the input viral DNA is integrated into the cell genome [33]. Since many human cells are semi-permissive to SV40 infection, the viral genome replicates poorly in these cells. Consequently, the few replicated DNA molecules remain in the episomal state, or are even lost, in a fraction of the cells [26]. Another mechanism of transformation, the paracrine mechanism, exerted by the SV40 Tag has been revealed in murine and canine cells. Indeed, it has been shown that the insulin-like growth factor type I (IGF-I) and hepatocyte growth factor (HGF) are secreted in SV40-positive cells [162,163] and may stimulate proliferation/transformation. Similarly, in human mesothelial cells SV40 Tag activates an autocrine/paracrine loop, involving the hepatocyte growth factor (HGF) and its cellular receptor, which is the product of the oncogene c-met [31], as well as the vascular endothelial growth factor (VEGF) and its cellular receptor VEGFR [165,166]. HGF and VEGF, released from SV40-positive human cells, bind their receptors in neighboring and distant SV40-positive and SV40-negative cells, driving them into proliferation and tumorigenesis. In this human cell model only one cell out of 100/1000 needs to express the Tag to transform all the cells of the monolayer [31,165,166].

Suggestive data are available on the role of SV40 Tag in the pathogenesis of human mesothelioma: (i) its ability to bind in vivo p53 and RB family proteins in human mesothelioma samples [167,168]; (ii) activation of Notch-1, a gene promoting cell cycle progression and cell proliferation, in primary human mesothelial cells [169]; (iii) induction of apoptosis in mesothelioma cells transfected with antisense DNA to the SV40 early region gene [170]; (iv) the presence of SV40 Tag-specific cytotoxic T lymphocytes in sera of patients affected by mesothelioma [171]; and (v) the poorer prognosis of mesotheliomas harboring SV40 early region sequences compared to SV40-negative mesotheliomas [172]. Moreover, mesothelial cells are particularly susceptible to infection and transformation by SV40 [30,31]. Asbestos, which is the main cause of human mesothelioma, cooperates with SV40 in transformation of murine cells as well as of human fibroblasts and mesothelial cells [30,173,174], suggesting that SV40 and asbestos may be co-carcinogens in the onset of the mesothelioma. Fluorescent in situ hybridization analysis has indicated that the RB and cyclin E/CDK2 genes undergo the same type of deregulation during the cell cycle in asbestos treated and SV40-transformed human mesothelial cells as well as in mesothelioma cells [175]. Recently, it has been shown that SV40 tumor antigens induce telomerase activity in human mesothelial cells, but not in human fibroblasts [174], suggesting that both SV40 oncoproteins specifically participate in the immortalization of mesothelial cells during mesothelioma development.

## Conclusion

The problems related to SV40 infection in the human population and to SV40 contribution to human cancer have been summarized in the recent evaluation of the "Immunization Safety Review Committee" established by the Institute of Medicine of the National Academies [176]. The Committee stated that "the evidence is inadequate to accept or reject a causal relationship between SV40-containing polio vaccines and cancer". In fact, the epidemiological studies conducted in the past are flawed by the difficulty in establishing which individuals received contaminated vaccines, in determining the dosage of infectious SV40 present in different lots of vaccine (due to formalin inactivation of poliovirus which may have variably affected SV40 infectivity), and finally in observing large cohorts of subjects for several decades after virus exposure to monitor for cancer development [155]. The Committee concluded that "the biological evidence is strong that SV40 is a transforming virus, but it is of moderate strength that SV40 exposure from polio vaccine is related to SV40 infection in humans and that SV40 exposure could lead to cancer in humans under natural conditions". The Committee also recommended the development of specific and sensitive serologic tests for SV40 and the use of standardized techniques that should be accepted and shared by all laboratories involved in SV40 detection. Although it may seem somehow a premature effort, the conviction that SV40 is implicated as a cofactor in the etiology of some human tumors has prompted programs to prepare a vaccine against the main viral oncoprotein, the SV40 Tag [177]. A recombinant vaccinia vector containing a safety-modified SV40 Tag sequence has been constructed [177]. Such a modified Tag excludes the p53 and RB protein binding sites as well as the amino-terminal oncogenic CRI and J domains [38], but preserves the immunogenic regions. Tumorigenesis studies carried out in vivo indicate that this vector can efficiently prime the immune response to provide effective, antigen specific prophylactic and therapeutic protection against SV40 Tag-expressing lethal tumors [178].

## Authors' contributions

FM wrote the initial draft of the manuscript. AC reviewed the manuscript integrating parts of the text. VB, CP and SS gave their contributions providing comments for various sections of the text including the references. MT had the primary responsibility for rewriting, incorporating comments, answering to the reviewers and editing the final text of the revised manuscript. All authors provided comments of various drafts, participated in direction setting discussions and have read and approved the final version.
